# Phylogenetic analysis and screening of elite cultivated *Ganoderma* strains in China

**DOI:** 10.1371/journal.pone.0360043

**Published:** 2026-10-23

**Authors:** Rujia Liang, Chaoan He, Keling Liu, Yuhao Zhang, Shuyu Han, Xiaofen Li, Ye Fu, Yuan Ren, Odeshnee Naicker, Chunlan Zhang

**Affiliations:** 1 College of Landscape Architecture, Changchun University, Changchun, Jilin, China; 2 Corn Research Institute, Changchun Academy of Agricultural Sciences, Changchun, Jilin Province, China; 3 Department of Plant and Soil Sciences, Faculty of Science, Engineering and Agriculture, University of Venda, Thohoyandou, South Africa; University of the Pacific, UNITED STATES OF AMERICA

## Abstract

*Ganoderma* cultivation has a long history in China, with *Ganoderma lingzhi*, now taxonomically recognized as *G. sichuanense*, being the primary cultivated species. Cultivation spans a wide range of climatic zones, from tropical to frigid regions. However, the use of diverse strains across regions has led to significant variability in growth traits and quality. To screen high-quality strains applicable to large-scale cultivation, we collected and evaluated 18 *Ganoderma* strains from core cultivation regions across Northeast, Central, Southwest and South China; this sampling panel covering the primary genetic and geographical diversity of cultivated *Ganoderma* nationwide. Species identification using both morphological and molecular methods revealed that 11 strains belonged to *G. sichuanense*, while 7 were identified as *G. sessile*. These results indicate that some strains currently cultivated as *G.* *sichuanense* may actually represent *G. sessile*, highlighting the importance of accurate species identification in cultivation practices. Growth evaluation showed that *G. sessile* strains generally exhibited shorter growth cycles than *G. sichuanense* strains, suggesting differences in growth performance interspecific. Chemical composition analysis showed that strain HZ-PJ (a strain of *G. sichuanense*) had the highest polysaccharide content (12.72 mg/g) and strongest DPPH antioxidant activity (3.0 μmol Trolox/g) among all tested strains. In contrast, strain HZ-TS (a strain of *G. sessile*) exhibited the highest levels of triterpenoids (12.2 mg/g) and flavonoids (10.0 mg/g). Collectively, strains HZ-PJ (*G. sichuanense*) and HZ-TS (*G. sessile*) combine favorable growth rates, high bioactive compound contents, and strong antioxidant potential, showing preliminary potential for standardized cultivation in northeastern China. It should be noted that this evaluation is only based on indoor culture assessments, and additional multi-location field trials focusing on yield stability, disease resistance and biological efficiency are still required to verify their commercial cultivation suitability. This study provides a systematic framework for strain evaluation and practical guidance for mushroom growers to select high-quality *Ganoderma* strains with stable agronomic traits and prominent functional activity.

## 1. Introduction

*Ganoderma* P. Karst., a genus belonging to the family Polyporaceae, has long been documented for its medicinal properties. The earliest written record of *Ganoderma* can be traced back to the ancient Chinese medical text *Shennong Bencao Jing*, in which the dried fruiting bodies of red and purple *Ganoderma* species are recorded as tonic and restorative materials in traditional Chinese medicine. *Ganoderma* is mainly distributed in China, Korea, and other Asian regions and has been highly valued for its health-beneficial effects for thousands of years. Its medicinal efficacy was officially recognized in the 2000 edition of the *Chinese Pharmacopoeia* [1,2].

Both the spores and fruiting bodies of *Ganoderma* species contain bioactive compounds, including polysaccharides, triterpenoids, proteins, amino acids, vitamins, alkaloids, and fatty acids [3,4]. These bioactive components exert a wide range of pharmacological activities, such as antitumor effects, immunomodulation, anti-fatigue activities, sleep improvement, anti-aging capacity, blood glucose regulation and liver protection [5–7]. Owing to its outstanding therapeutic prospects, *Ganoderma* has achieved tremendous commercial value; the global market size of *Ganoderma*-derived products exceeded 2.5 billion US dollars in 2013, with China, Japan and South Korea serving as the major producers and exporters [8].

The mainstream artificially cultivated taxon is *Ganoderma sichuanense* (formerly referred to as *Ganoderma lingzhi*) [9–11], which is grown across a broad climatic range, from tropical and subtropical to temperate regions [12]. By contrast, *G. sessile* spreads extensively in China’s wild forests but has not realized large-scale commercial cultivation, with only scattered small trial planting cases reported. From an economic perspective, *G. sichuanense* occupies a dominant position in domestic *Ganoderma* industry supported by mature industrial chains; wild *G. sessile* has no direct commercial production value, but its unique genetic background and differential metabolite profiles make it a valuable wild germplasm for breeding. Multiple strains of *G. sichuanense* are currently applied in artificial cultivation, yet their agronomic performances and finished product quality differ drastically [13,14].

Carrying out parallel comparison of the two species has both scientific and commercial value: scientifically, it fills the research gap of systematic interspecific comparison between industrial cultivated *Ganoderma* and wild germplasm, and reveals species-specific differences in growth cycle and metabolite enrichment; commercially, multi-index evaluation can identify the cultivation bottlenecks and germplasm advantages of *G. sessile*, providing theoretical basis for its subsequent domestication and strain improvement, as well as reference data for optimizing standardized cultivation of *G. sichuanense*. Nevertheless, few previous studies have set up cross-species parallel evaluations of these two taxa [15–17].

Existing germplasm evaluation systems have three obvious limitations: first, most assessments rely solely on single biochemical or morphological indicators, without integrating molecular phylogenetic identification, multi-dimensional growth indicators and multi-component activity detection; second, sampling campaigns are restricted to isolated local cultivation areas, failing to cover germplasm resources across nationwide core producing zones; third, few studies simultaneously compare the mainstream cultivated species *G. sichuanense* and widely distributed *G. sessile*, ignoring interspecific disparities in growth cycle and metabolite enrichment [18–22]. These unresolved research gaps highlight the need for the present multi-strain, cross-species and systematic multi-index assessment. Therefore, it is urgent to screen and popularize elite strains of both *G. sichuanense* and *G. sessile* to support standardized domestic cultivation.

In this study, 18 *Ganoderma* strains including *G. sichuanense* and *G. sessile* were collected from diverse ecological regions in China. These strains were first identified to the species level using a combination of morphological characterization and molecular techniques. Subsequently, their cultivation adaptability and yield stability were comprehensively evaluated under controlled conditions. Based on the results, we provide practical suggestions for strain selection and cultivation for commercial growers [23–27].

## 2. Materials and methods

### 2.1. Sample collection, transportation, and aseptic processing

A total of 18 strains were collected from multiple forest and cultivation zones across China following unified sampling criteria. Pure culture strains were directly provided and preserved by the person in charge of each sampling site during our field collection. In contrast, strains labeled as wild isolates were newly obtained in our lab via tissue isolation and purification from fresh wild *Ganoderma* fruiting bodies. All materials cover two categories: commercial breeding pure cultures and wild germplasm resources, with 2–4 strains obtained per geographic region. The strain selection rationale focused on two target species, *G. sichuanense* and *G. sessile*.

All samples were transported to the laboratory under sterile and temperature-controlled conditions immediately after collection. Fruiting bodies were individually wrapped in sterile aluminum foil or placed in sterile sampling bags and kept in insulated containers with ice packs (4 °C) to minimize microbial contamination and metabolic changes during transit. Pure culture strains obtained from culture collections were transported according to standard microbial preservation protocols.

Upon arrival at the laboratory, samples were processed within 24 h. Fresh fruiting bodies were surface-sterilized using 75% ethanol followed by rinsing with sterile distilled water before tissue isolation. Small internal tissue fragments were aseptically excised and transferred onto potato dextrose agar (PDA) plates supplemented with antibiotics (e.g., streptomycin or ampicillin) to suppress bacterial growth.

All cultures were incubated at 25 °C and regularly monitored for contamination. Single colonies were subcultured repeatedly to obtain pure isolates. Morphological uniformity and microscopic examination were used to confirm culture purity. In addition, three barcodes (ITS, RPB2, TEF1-α) were used for multilocus molecular identification to confirm strain identity and species assignment.

For short-term storage, purified cultures were maintained on PDA slants at 4 °C. For long-term preservation, cultures were stored in 20% glycerol stocks at −80 °C. All handling procedures were carried out under aseptic conditions in a laminar flow hood to ensure culture integrity prior to subsequent analyses [26].

#### 2.1.1 Test strains.

Eighteen *Ganoderma* strains were collected from various cultivation regions across China. Details of each strain, including collection site, strain source, and collection date, are presented in Table 1, Voucher specimen numbers follow the format HCCUP-XXXXX. All dried fruiting-body vouchers and fixed agar plate specimens are deposited in the Herbarium of Plant Pathology, Changchun University (abbreviated HCCUP). All living fungal cultures were preserved in the Changchun University Fungal Culture Collection (CCUCC), with official culture accession numbers prefixed CCUCC. Fig 1 displays the map overview of each sampling point.

**Table 1:** Information of tested *Ganoderma* strains.

Strains	Voucher specimen number	Culture collection accession numbers	Collection site (city, province)	Strain Source	Latitude (°N)	Longitude (°E)
*Ganoderma* sp. (HZ-YB)	HCCUP 26-(001–099)	CCUCC 01947	Wuhan, Hubei	pure cultures	30.59	114.31
*Ganoderma* sp. (HZ-MD)	HCCUP 27-(001–099)	CCUCC 01948	Wuhan, Hubei	pure cultures	30.59	114.31
*Ganoderma* sp. (HZ-PJ)	HCCUP 28-(001–099)	CCUCC 01949	Wuhan, Hubei	pure cultures	30.59	114.31
*Ganoderma* sp. (LJ-1)	HCCUP 29-(001–099)	CCUCC 01950	Wuhan, Hubei	pure cultures	30.59	114.31
*Ganoderma* sp. (HZ-TS)	HCCUP 30-(001–099)	CCUCC 01951	Wuhan, Hubei	pure cultures	30.59	114.31
*Ganoderma* sp. (HZ-CZ)	HCCUP 31-(001–099)	CCUCC 01952	Zhumadian, Henan	pure cultures	32.99	114.03
*Ganoderma* sp. (WH-CZ)	HCCUP 32-(001–099)	CCUCC 01953	Wuhan, Hubei	pure cultures	30.59	114.31
*Ganoderma* sp. (CLZ-1)	HCCUP 33-(001–099)	CCUCC 01954	Xuzhou, Jiangsu	pure cultures	34.22	117.16
*Ganoderma* sp. (22GL-13)	HCCUP 34-(001–099)	CCUCC 01955	Zhengzhou, Henan	pure cultures	34.75	113.67
*Ganoderma* sp. (22GL-12)	HCCUP 35-(001–099)	CCUCC 01956	Daxinganling, Heilongjiang	isolate them from fruiting bodies(wild)	50.75	124.63
*Ganoderma* sp. (22GL-9)	HCCUP 36-(001–099)	CCUCC 01957	Chengdu, Sichuan	isolate them from fruiting bodies(wild)	30.67	104.07
*Ganoderma* sp. (22GL-6)	HCCUP 37-(001–099)	CCUCC 01958	Chengdu, Sichuan	pure cultures	30.67	104.07
*Ganoderma* sp. (41GL-3)	HCCUP 38-(001–099)	CCUCC 01959	Jilin, Jilin	isolate them from fruiting bodies(wild)	43.85	126.53
*Ganoderma* sp. (41GL-8)	HCCUP 39-(001–099)	CCUCC 01960	Baishan, Jilin	isolate them from fruiting bodies(wild)	42.02	127.46
*Ganoderma* sp. (41GL-5)	HCCUP 40-(001–099)	CCUCC 01961	Jinan, Shandong	pure cultures	36.68	117.00
*Ganoderma* sp. (41GL-13)	HCCUP 41-(001–099)	CCUCC 01962	Yizhou, Shanxi	isolate them from fruiting bodies(wild)	35.20	110.90
*Ganoderma* sp. (41GL-2)	HCCUP 42-(001–099)	CCUCC 01963	Longquan, Zhejiang	isolate them from fruiting bodies(wild)	28.07	119.16
*Ganoderma* sp. (41GL-4)	HCCUP 43-(001–099)	CCUCC 01964	Longquan, Zhejiang	pure cultures	28.07	119.16

**Fig 1:**
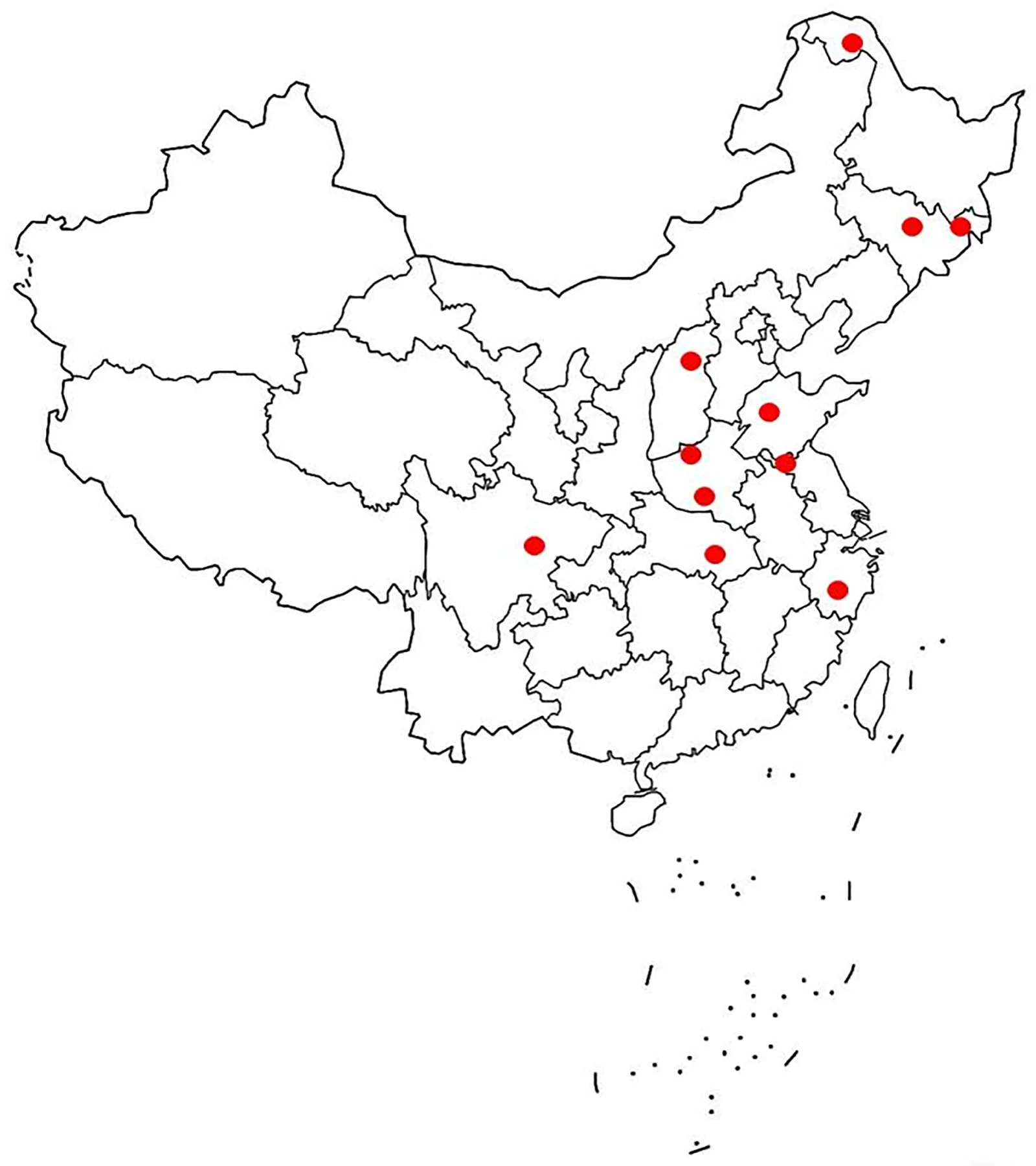
Overview map of Ganoderma sampling sites. Red dots represent the strain collection location.

### 2.2 Study on phylogeny of tested strains

#### 2.2.1 DNA extraction.

Mycelia were harvested by scraping the surface of actively growing cultures using a sterile inoculation spatula and transferring them into potato dextrose broth (PDB). Cultures were incubated at 25 °C with shaking at 150 rpm for 3–4 days until sufficient mycelial biomass was obtained. The mycelia were filtered using sterile gauze, transferred onto sterile filter paper, and air-dried. The dried samples were frozen in liquid nitrogen and ground into a fine powder using a sterile mortar and pestle. Genomic DNA was extracted using the Nuclear Plant Genomic DNA Kit (Changchun Feikai Biological Co., Ltd.) following the manufacturer’s protocol. Extracted DNA was stored at −20 °C for subsequent analysis [29,30].

#### 2.2.2 Polymerase chain reaction.

To determine the phylogenetic relationships among the tested *Ganoderma* strains, three genetic loci were targeted for amplification: the internal transcribed spacer (ITS) region, the RNA polymerase II second largest subunit (RPB2) gene, and the translation elongation factor 1-alpha (TEF1-α) gene. Each gene region was amplified using specific primer pairs and optimized thermal cycling conditions to ensure high specificity and yield.

Amplification of the ITS region was carried out using the primer pair ITS4/ITS5. The PCR reaction began with an initial denaturation at 95 °C for 3 minutes to fully separate the DNA strands. This was followed by 34 cycles of denaturation at 94 °C for 40 seconds, annealing at 54 °C for 45 seconds to allow primer binding, and extension at 72 °C for 1 minute for DNA synthesis. A final extension was performed at 72 °C for 10 minutes to ensure complete amplification of all fragments, and the products were held at 4 °C until further analysis.

For the RPB2 gene, amplification was performed using the primer pair 5F/7CR. The reaction conditions included an initial denaturation at 95 °C for 1 minute, followed by 34 cycles consisting of denaturation at 95 °C for 1 minute, annealing at 58 °C for 45 seconds, and extension at 72 °C for 1.5 minutes. A final extension step at 72 °C for 10 minutes was used to ensure complete synthesis of the target amplicons, after which the samples were stored at 4 °C.

The TEF1-α gene was amplified using the primer pair 983F/1567R. The PCR reaction began with an initial denaturation at 95 °C for 3 minutes to fully separate the DNA strands. This was followed by 34 cycles of denaturation at 94 °C for 40 seconds, annealing at 56 °C for 45 seconds to allow primer binding, and extension at 72 °C for 1 minute for DNA synthesis. A final extension was performed at 72 °C for 10 minutes to ensure complete amplification of all fragments, and the products were held at 4 °C until further analysis.

#### 2.2.3 Agarose gel electrophoresis, detection and sequence analysis.

The PCR amplification products were verified by 1% agarose gelelectrophoresis and subsequently sent to Beijing Qingke Biotechnology Co., Ltd. (Beijing, China) for sequencing. To prepare a 1% agarose gel, 1 g of agarose was dissolved in 100 mL of 1 × Tris-Borate-EDTA (TBE) buffer. Two microliters of M5 HiPure Next III GelRed (a nucleic acid stain) was added to the solution, which was then microwaved for approximately one minute until fully dissolved. The hot agarose solution was poured into a gel tray fitted with a five-well comb (model 1-S5) and left at room temperature to solidify before use [31,32]. A molecular weight marker (5 µL) was loaded into the first well to serve as a size reference. Subsequently, 5 µL of each PCR product was loaded into separate wells, with the loading order carefully recorded.

Electrophoresis was carried out at 110 V until the dye front had migrated an appropriate distance through the gel. The power was then turned off, and the gel was carefully removed using insulated gloves. It was transferred to a gel imaging system for visualization under UV light. Bands corresponding to the expected sizes of the amplified loci (ITS, RPB2, and TEF1-α) were identified and used to select samples for sequencing.

PCR products with clear, single bands were chosen for sequencing. A volume of 20 µL of the selected amplicon was transferred into a clean, labeled centrifuge tube. Additionally, 5 µL each of the forward and reverse primers was prepared in separate labeled tubes for submission. DNA sequencing was outsourced to Changchun Biotechnology Engineering Co. (Changchun, Jilin, China). The obtained sequences were used for subsequent phylogenetic analyses [33–35].

### 2.3 Phylogenetic tree construction

Prior to phylogenetic analysis, raw sequences were quality-controlled with FastQC v0.11.9, and low-quality reads and adapter sequences were trimmed with Trimmomatic v0.39. Cleaned reads were de novo assembled into consensus sequences using SPAdes v0.15.4. Consensus sequences were then used for BLASTn searches against the NCBI nr/nt database to verify taxonomic identities and avoid cross-contamination. Sequences were selected from GenBank according to specific criteria: belonging to the genus *Ganoderma*, with a length >500 bp and sequence similarity >95% to the query sequences. Details of the selected fungal strains and GenBank accession numbers are provided in Table 2. The sequences of ITS, RPB2, and TEF1 were concatenated using PhyloSuite v1.2.2. Phylogenetic analyses were performed using Phylosuite. ModelFinder was used to determine the optimal nucleotide substitution model, and the TN93 + G + I model was selected for all loci. Coriolopsis sp. Dal 10786 was used as the outgroup, and maximum likelihood (ML) trees were constructed in Phylosuite with 1,000 bootstrap replicates for branch support assessment, and the scale bar (0.05 substitutions per site) indicates the degree of genetic change, where longer branches represent greater sequence divergence. Bootstrap support values of 85% or greater for ML and 0.95 or greater for PP are indicated at the nodes as ML/PP, reflecting the confidence in each branching event [36,37].

**Table 2:** Specimen information and GenBank accession numbers used in the phylogenetic analysis.

Species	Strains	Geographic Origin	GenBank Accession No.			Reference
			RPB2	TEF1	ITS	
*Ganoderma resinaceum*	MUCL 52253	France	MK554764	MK554737	MK554786	(Cabarroi-Hernández et al. 2019)
*Ganoderma resinaceum*	CBS 194.76	Netherlands	–	KJ143934	–	(Zhou et al. 2015)
*Ganoderma sessile*	Dai 16403	USA	MZ345739	MZ221689	MZ3MK55473754934	(Sun et al. 2022)
*Ganoderma resinaceum*	LGAM 565	Greece	MG837823	MG837860	MG706252	(Grinn-Gofroń et al. 2021)
*Ganoderma lucidum*	BEOFB 432	Serbia	KX371601	KX371598	KX371595	(Ćilerdžić et al. 2018)
*Ganoderma lucidum*	BEOFB 431	Serbia	KX371600	KX371597	KX371594	(Ćilerdžić et al. 2018)
*Ganoderma resinaceum*	CCBAS	Athens, Attiki	MG837814	MG837851	MG706242	(Ćilerdžić et al. 2018)
*Ganoderma resinaceum*	LGAM 486	Athens, Attiki	MG837822	MG837859	MG706251	(Ćilerdžić et al. 2018)
*Ganoderma sessile*	JV 1209/27	AZ, USA	KJ143976	KJ143937	KF605630	(Zhou et al. 2015)
*Ganoderma flexipes*	Dai 20461	China	MZ245403	MZ221657	MZ354925	(Sun et al. 2022)
*Ganoderma flexipes*	Cui 13863	China	MZ245402	MZ221656	MZ354924	(Sun et al. 2022)
*Ganoderma tropicum*	Dai 19491	Sri Lanka	MZ358827	MZ221709	MZ354901	(Sun et al. 2022)
*Ganoderma hoehnelianum*	Cui 13904	China	MZ245411	MZ221666	MZ354935	(Sun et al. 2022)
*Ganoderma tropicum*	Dai 20029	China	MZ358826	MZ221708	MZ354902	(Grinn-Gofroń et al. 2021)
*Ganoderma lingzhi*	MFLU 19–2193	China	MN423128	MN423161	MN396323	(Jargalmaa et al. 2017)
*Ganoderma lingzhi*	MFLU 19–2192	China	MN423127	MN423160	MN396322	(Jargalmaa et al. 2017)
*Ganoderma multipileum*	Dai 17569	China	MZ345733	MZ221676	MZ354896	(Sun et al. 2022)
*Ganoderma tropicum*	Dai 19679	China	MZ358825	MZ221707	MZ354900	(Sun et al. 2022)
*Ganoderma mutabile*	Dai 20414	China	MZ345735	MZ221680	MZ354977	(Sun et al. 2022)
*Ganoderma sichuanense*	Dai 19651	China	MZ345742	MZ221693	MZ354929	(Sun et al. 2022)
*Ganoderma leucocontextum*	JZB2114015	China	MH294411	MH294378	MH294311	(Adotey et al. 2026)
*Ganoderma sinense*	JZB2114021	China	MH294416	MH294383	MH294316	(Adotey et al. 2026)
*Ganoderma curtisii*	CBS 100131	USA	KJ143966	KJ143926	JQ781848	(Zhou et al. 2015)
*Ganoderma shanxiense*	Dai 18921	China	MZ345740	MZ221691	MZ354909	(Sun et al. 2022)
*Ganoderma destructans*	Dai 16431	China	MG367512	MG367569	MG279177	(Sun et al. 2022)
*Ganoderma martinicense*	He 2240	USA	MG367503	MG367557	MG279163	(Xing et al. 2018)
*Ganoderma philippii*	Cui 14443	Hainan, China	MG367524	MG367578	MG279188	(Xing et al. 2018)
*Ganoderma tsugae*	AFTOL-ID 771	USA	DQ408116	DQ059048	DQ206985	(Brandon Matheny et al. 2007)
*Ganoderma oregonense*	JZB2114013	China	MH294409	MH294376	MH294309	(Adotey et al. 2026)
*Ganoderma applanatum*	Cui 14121	China	OK665319	OK665208	OK642190	–
G*anoderma hoehnelianum*	Yuan 6337	China	MG367498	MG367551	MG279160	(Xing et al. 2018)
*Ganoderma weberianum*	Dai 19682	China	MZ358830	MZ221713	MZ354932	(Sun et al. 2022)
*Ganoderma weberianum*	Dai 19673	China	MZ358829	MZ221712	MZ354930	(Sun et al. 2022)
*Ganoderma applanatum*	Cui 14070	China	MZ245387	MZ221636	MZ354914	(Sun et al. 2022)
*Ganoderma tsugae*	Cui 14112	China	MG367534	MG367587	MG279196	(Xing et al. 2018)
*Ganoderma tsugae*	Cui 14110	China	MG367533	MG367586	MG279195	(Xing et al. 2018)
*Ganoderma tsugae*	Dai 15529	China	MG367536	MG367590	MG279197	(Xing et al. 2018)
*Coriolopsis* sp.	Dai 10786	China	KX885089	KX838442	KC867376	–
*Ganoderma lingzhi*	Cui 14342	China	MG367517	MG367571	MG279179	(Xing et al. 2018)
*Ganoderma lingzhi*	Cui 14375	China	MG367518	MG367572	MG279180	(Xing et al. 2018)
*Ganoderma* sp.	22GL-9	China	PV610823	PV610806	PV583440	This study
*Ganoderma* sp.	22GL13	China	PV610824	PV610807	PV583441	This study
*Ganoderma* sp.	22GL-12	China	PV610825	PV610808	PV583442	This study
*Ganoderma* sp.	41GL-5	China	PV610829	PV610812	PV583446	This study
*Ganoderma* sp.	41GL-2	China	PV610830	PV610813	PV583447	This study
*Ganoderma* sp.	WH-CZ	China	PV610831	PV610814	PV583448	This study
*Ganoderma* sp.	41GL-13	China	PV610833	PV610816	PV583450	This study
*Ganoderma* sp.	41GL-8	China	PV610834	PV610817	PV583451	This study
*Ganoderma* sp.	HZ-PJ	China	PV610835	PV610818	PV583452	This study
*Ganoderma* sp.	HZ-CZ	China	PV610838	PV610821	PV583455	This study
*Ganoderma* sp.	41GL-3	China	PV610827	PV610810	PV583444	This study
*Ganoderma* sp.	HZ-YB	China	PV610822	PV610805	PV583439	This study
*Ganoderma* sp.	LJ-1	China	PV610836	PV610819	PV583453	This study
*Ganoderma* sp.	CLZ-1	China	PV610837	PV610820	PV583454	This study
*Ganoderma* sp.	41GL-4	China	PV610832	PV610815	PV583449	This study
*Ganoderma* sp.	HZ-MD	China	PV610828	PV610811	PV583445	This study
*Ganoderma* sp.	HZ-TS	China	PV610826	PV610809	PV583443	This study
*Ganoderma* sp.	22GL-6	China	PZ454104	PZ454105	PZ384670	This study

### 2.4 Morphological identification

To evaluate morphological characteristics, *Ganoderma* strains were cultivated on linden wood segments. Logs approximately 4–7 cm in diameter and 25–30 cm in length were bundled into ~30 cm bundles using cotton rope, placed in polypropylene bags, and sterilized by autoclaving. After cooling, the logs were inoculated with fungal strains.

Once colonized by *Ganoderma* mycelia, the logs were transferred to a cold shed located at the West Campus of Changchun University (Changchun City, Jilin Province) in early May, where ambient temperatures ranged from 20°C to 25°C. Cultivation was managed under conventional mulching conditions. Fruiting body development was monitored, and morphological features of the basidiocarps and spores were subsequently documented [34,35].

#### 2.4.1 Fruiting body morphology.

Fruiting bodies were gently cleaned to remove surface debris without damaging structural features. Morphological characteristics such as cap shape, surface texture, color, pore structure, and stipe formation were observed and recorded for each strain.

#### 2.4.2 Microscopic morphology.

After surface impurities were removed, the fruiting bodies of *Ganoderma* lucidum were naturally air-dried and cut transversely and longitudinally to obtain uniform thin slices. The slices were mounted on scanning electron microscope (SEM) stubs with conductive carbon films, followed by gold sputtering using an ion sputtering coater for 90 s at a vacuum of 10 Pa and a sputtering current of 15 mA to form an ultrathin conductive gold film and suppress charging effects.

To ensure the quality of viewing surfaces, the slices were preliminarily screened using a Nikon Ti2-U upright fluorescence microscope (Nikon, Japan). Only flat and intact specimens were selected for subsequent SEM observation. Microscopic observation was performed using a TESCAN VEGA 3 scanning electron microscope (TESCAN, Czech Republic). Images were acquired in secondary electron mode at a medium scanning speed with the following parameters: accelerating voltage of 15 kV, working distance of 8–10 mm, and spot size of 3.0. Image quality was optimized by adjusting brightness and contrast and performing astigmatism correction. The cross-sectional micro-morphologies, including hyphal structure and spore morphology, were observed and recorded at multiple magnifications.

### 2.5 Growth rate and field cultivation of test strains

#### 2.5.1 Mycelial growth rate measurement.

Potato Dextrose Agar (PDA) medium was prepared and sterilized via autoclaving, then poured into Petri dishes under sterile conditions (10–15 mL per plate). Once cooled, each test strain was aseptically inoculated onto the PDA. For each strain, five plates were inoculated (forming one replicate group), and a total of 18 strain groups were prepared. Plates were incubated at 25°C for approximately 7 days. Colony development was monitored daily, while colony diameter was measured at approximately 3–4-day intervals, resulting in two to three measurements during the observation period. Because the tested strains differed in growth rate, some fast-growing strains reached the edge of the Petri dishes earlier than the slower-growing strains. Measurements were continued until the colonies reached the edge of the plates, and the final colony diameter was recorded at that point. Colony diameter was measured using the cross-measurement method, and the mycelial growth rate was calculated in cm/day using the following equation:


$$\begin{array}{c} \text{Growth rate }(\text{cm}/\text{day})=\frac{{\text{D}}_{\text{t}}-{\text{D}}_{\text{0}}}{\text{t}} \end{array}$$

where D_t_ represents the final colony diameter, D_0_ represents the diameter of the initial inoculum plug, and t represents the total incubation period. Mycelial morphology was also recorded photographically.

#### 2.5.2 Field cultivation experiment.

Colonized *Ganoderma* mycelia were transferred to mushroom grow bags (10 × 23 × 38 cm), each filled with 1.6 kg of sterilized substrate composed of 90% sawdust, 5% wheat bran, 4% corn flour, and 1% lime. After inoculation and full colonization, the bags were moved to a greenhouse maintained at 20°C–32°C and 80%–90% humidity for casing cultivation. Each strain was cultivated in 15 replicate grow bags under standardized conditions. Throughout the fruiting period, key developmental stages, including primordia formation, pinning, differentiation, and maturation, were carefully observed and documented through photography. Upon full maturation, the morphology and yield of the fruiting bodies were assessed. Several morphological and production parameters were measured for each strain. The number of branches was defined as the occurrence of multiple caps emerging from a single stipe. Cap diameter was measured at its widest transverse point, while cap thickness was assessed at the cap's edge. Stipe length was measured from the base of the fruiting body to the point of cap attachment. Finally, the total fresh weight of fruiting bodies harvested per bag was recorded. For each parameter, the average value was calculated across all 15 replicates to ensure robust and representative data.

### 2.6 Determination of bioactive components

#### 2.6.1 Sample preparation.

Freshly harvested *Ganoderma* fruiting bodies were first cleaned by gently brushing off surface soil and dust. They were then oven-dried at 40 °C for 24 hours until a constant weight was achieved. The dried material was chopped into small pieces, ground into a fine powder using a mechanical grinder, and passed through a 20-mesh sieve. The resulting powder was stored in a desiccator until further analysis.

#### 2.6.2 Polysaccharide content.

Total polysaccharide content was determined using a commercial detection kit (Product No. ZDT-2-Y, Suzhou Komine Biotechnology Co., Ltd.). For extraction, 0.05 g of powdered sample was suspended in 1 mL of distilled water, homogenized, and heated in a 100 °C water bath for 2 hours. After cooling, the mixture was centrifuged at 10,000 rpm for 10 minutes, and the supernatant was collected. To remove non-polysaccharide impurities and small-molecular-weight soluble carbohydrates, a 0.2 mL aliquot of the supernatant was added to 0.8 mL of anhydrous ethanol, mixed thoroughly, and incubated overnight at 4 °C to precipitate polysaccharides. The precipitate was collected by centrifugation at 10,000 rpm for 10 minutes, re-dissolved in 1 mL of distilled water, and analyzed according to the kit instructions [36,38].

### 2.6.3 Antioxidant capacity

Antioxidant activity was assessed using DPPH and ABTS radical scavenging assays with commercial kits (Product Nos. DPPH-2-D and ABTS-2-D, Suzhou Komine Biotechnology Co., Ltd.). For extraction, 0.1 g of powdered sample was homogenized in 1 mL of extraction buffer in an ice bath and then centrifuged at 10,000 rpm at 4 °C for 10 minutes. The supernatant was retained for further testing as per the kit protocols [39,40,41].

The radical scavenging activity was calculated as follows:


$$\begin{array}{c} Scavenging\;activity(\%)=\frac{A_{\text{0}}-A_{\text{1}}}{A_{\text{0}}}\times \text{1}\text{0}\text{0} \end{array}$$

Where A0is the absorbance of the control and A1is the absorbance of the sample.

#### 2.6.4 Flavonoid content.

Flavonoid content was measured using a detection kit (Product No. LHT-2-G, Suzhou Komine Biotechnology Co., Ltd.). The sample was dried to constant weight, crushed, and sieved through a 35-mesh screen. A 0.02 g portion of the powder was extracted with 2 mL of the extraction buffer at 60 °C for 2 hours with constant shaking. The mixture was then centrifuged at 10,000 rpm at 25 °C for 10 minutes, and the supernatant was used for analysis in accordance with the manufacturer's instructions [37,42].

The total flavonoid content was calculated as follows:


$$\begin{array}{c} \text{Total flavonoid content}=\frac{\text{C}\times \text{V}}{\text{m}} \end{array}$$

where C is the concentration determined from the standard curve (mg/mL), V is the volume of the extract (mL), and m is the sample weight (g). The results were expressed as mg rutin equivalents per gram of dry weight (mg RE/g DW).

#### 2.6.5 Triterpene content.

For triterpene quantification, 0.1 g of *Ganoderma* powder was transferred into a 50 mL centrifuge tube containing 10 mL of anhydrous ethanol. After vortexing, the sample was ultrasonicated for 20 minutes and centrifuged at 7000 rpm for 10 minutes. A 0.4 mL aliquot of the supernatant was transferred to a 10 mL stoppered test tube and evaporated to dryness in a 90 °C oven. After cooling to room temperature, 0.2 mL of 5% vanillin–glacial acetic acid solution and 0.8 mL of perchloric acid were added. The mixture was vortexed and incubated in a 70 °C water bath for 15 minutes for color development. The reaction was then cooled in an ice bath for 5 minutes, followed by the addition of 5 mL of glacial acetic acid. After thorough mixing, the absorbance was measured at 546 nm [43].

The triterpene content was calculated as follows:


$$\begin{array}{c} \text{Triterpene content}=\frac{\text{C}\times \text{V}}{\text{10}\times \text{m}} \end{array}$$

where C is the concentration obtained from the standard curve (mg/mL), V is the extract volume (mL), and m is the sample weight (g). The results were expressed as mg oleanolic acid equivalents per gram of dry weight (% OAE DW).

#### 2.6.6 Trace elements and heavy metal analysis.

The concentrations of cadmium (Cd), lead (Pb), potassium (K), and zinc (Zn) in *Ganoderma* fruiting bodies were measured. For the analysis, 0.5 g of powdered sample was placed in a 50 mL digestion tube, moistened with 5–10 drops of distilled water, and left to stand for 1–2 minutes. Then, 5 mL of concentrated sulfuric acid was added, mixed, and allowed to stand for 5 minutes. Two drops of perchloric acid and several glass beads were added before digestion.The digestion procedure involved three heating stages: 90 °C for 10 minutes, 200 °C for 30 minutes, and 340 °C for 40 minutes, until the residue turned white or off-white. After cooling, the digested sample was transferred to a 50 mL volumetric flask, rinsed with distilled water, and diluted to 10 mL. The concentrations of heavy metals and trace elements were then determined using atomic absorption spectrometry [44].

### 2.7 Data analysis

Average values for each strain were calculated using Microsoft Excel for comparative analysis. For biochemical analyses (e.g., polysaccharide content, antioxidant capacity), all experiments were independently repeated in triplicate. Statistical analyses were conducted using Origin 2018 and IBM SPSS Statistics 26. One-way analysis of variance (ANOVA) was employed to determine statistically significant differences among treatments, with a significance threshold set at *p* < 0.05. Differences with *p* values below this threshold were considered statistically significant, indicating meaningful variation in growth characteristics or bioactive compound content among the *Ganoderma* strains evaluated. Using Duncan's new multiple range test for multiple comparison of data, and marking letters (p < 0.05) to conduct difference significance analysis.

## 3. Results

### 3.1 Phylogeny of tested strains

Phylogenetic identification of *Ganoderma* strains is critical for clarifying the species composition in cultivation regions, which provides a reliable basis for subsequent evaluation of their agronomic traits and functional components. Among the 18 tested *Ganoderma* strains, 11 (22GL-9, 41GL-2, 22GL-12, HZ-CZ, WH-CZ, 41GL-5, 41GL-13, 22GL-13, HZ-PJ, 41GL-8, 22GL-6) were identified as *G. sichuanense*. However, strains 41GL-5 and 22GL-13 obtained a bootstrap value of only 29% at a subordinate internal node, which merely indicates weak resolution of their intraspecific relatedness within the *G. sichuanense* clade, rather than casting doubt on their species-level classification. The node defining the entire *G. sichuanense* clade receives high bootstrap support, firmly verifying that these two isolates belong to *G. sichuanense*. The remaining 7 strains (41GL-4, HZ-CZ, CLZ-1, LJ-1, HZ-TS, HZ-YB, and HZ-MD) were classified as *G. sessile* (Fig 2). These results confirm that both species are present in cultivation regions traditionally associated with *G. sichuanense*.

**Fig 2:**
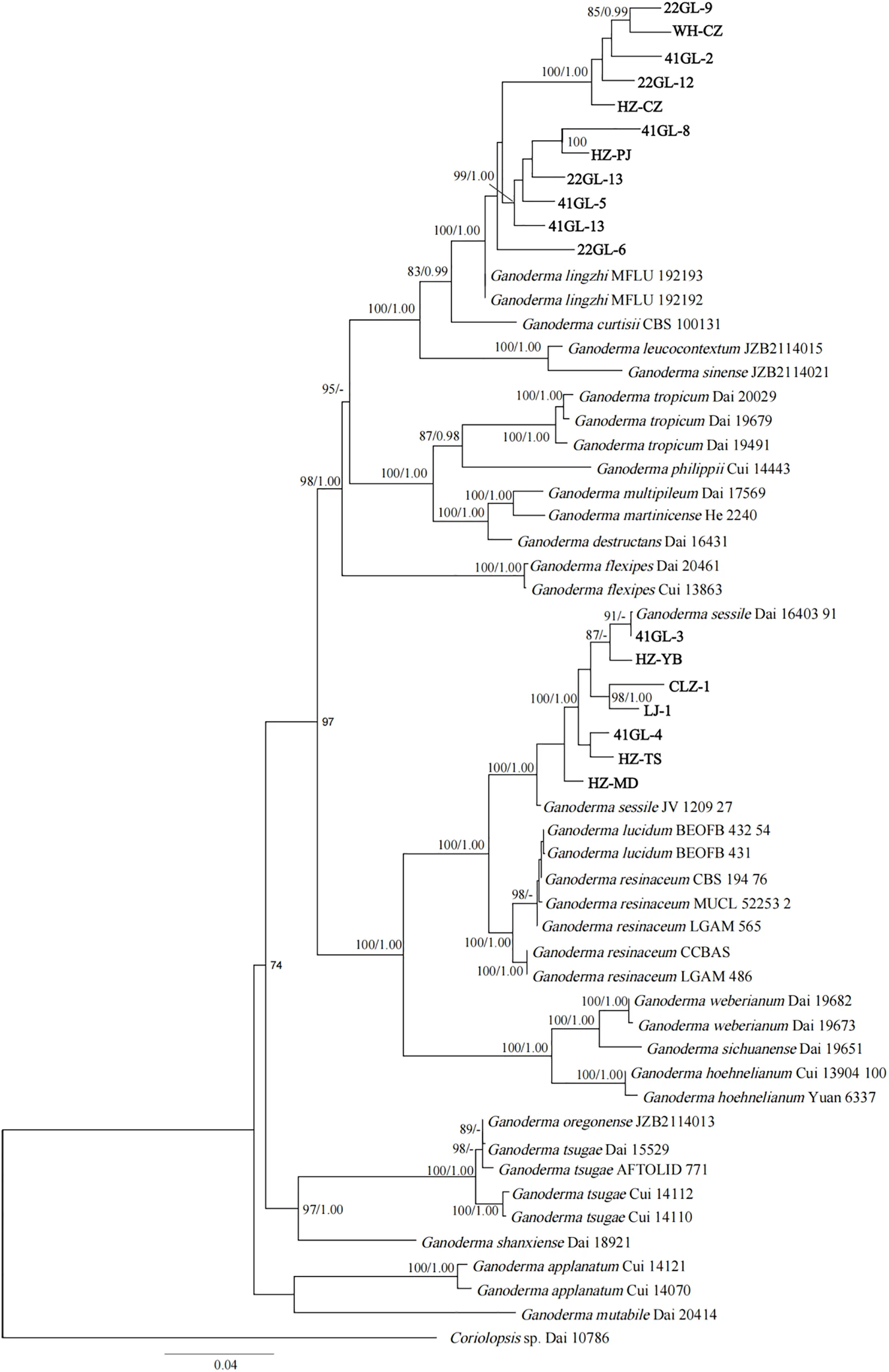
Phylogenetic tree of the tested *Ganoderma* strains based on *RPB2*, *EF1-a*, ITS joint construction, *Coriolopsis* sp. Dai 10786 was used as the outgroup. Bootstrap support values of 85%or greater for ML and 0.95 or greater for PP are indicated at the nodes as ML/PP.

### 3.2 Morphological characterization

Morphological analysis further supported species identification. The macroscopic and microscopic images in Fig 3 are representative photographs captured from the designated representative strains 41GL-5 (*G. sichuanense*) and HZ-TS (*G. sessile*), respectively. Morphological quantification was performed on multiple isolates of each species; however, no obvious intraspecific divergence in macroscopic and microscopic traits was observed, meaning the phenotypic characteristics of these two strains are representative of their respective species groups.

**Fig 3:**
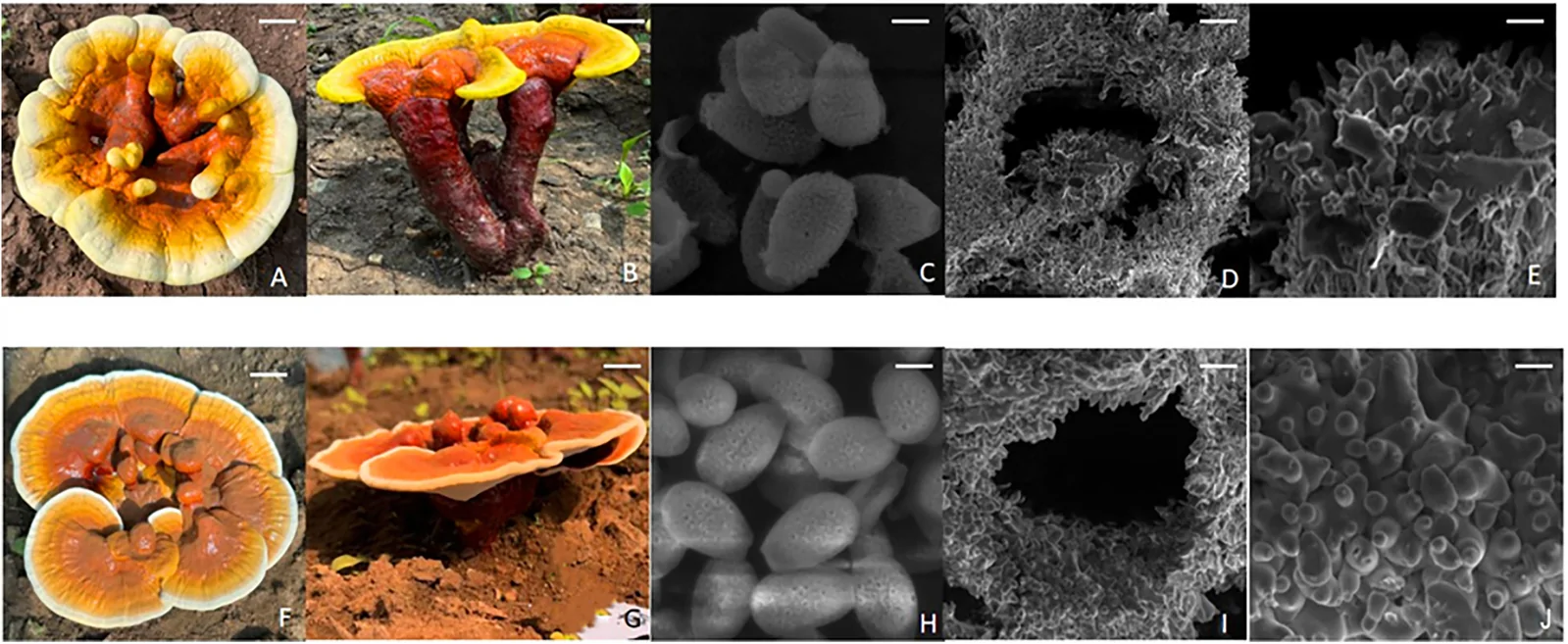
Comparison of microstructure between *G. sichuanense* and *G.sessile.* (A–B) Basidiocarp of *G. sichuanense,* showing cap and stipe (bar = 1 cm);(C) Basidiospores of *G. sichuanense* (bar = 2 μm);(D–E) Hymenophore tubes of *G. sichuanense* (D: bar = 100 μm; E: bar = 20 μm);(F–G) Basidiocarp of *G. sessile*, showing cap and stipe (bar = 1 cm);(H) Basidiospores of *G. sessile* (bar = 2 μm); (I–J) Hymenophore tubes of G. sessile (I: bar = 100 μm; J: bar = 20 μm).

Strains identified as *G. sichuanense* exhibited cap thicknesses of 2.5–3 cm and stipe lengths of 7–11 cm (Fig 3A–3B). Their spores were oval with smooth surfaces, measuring approximately 5.6–9.2 × 3.7–5.3 µm (Fig 2C). Hymenophore tubes were densely packed, slender, and tubular, 1–3 mm in length, and uniformly 0.5–1 mm in diameter with smooth inner walls (Fig 3D–3E).

In contrast, *G. sessile* strains showed cap thicknesses of 1.7–2.6 cm and shorter stipe lengths of 2–4 cm (Fig 3F–3G). Their spores were more rounded with noticeable surface wrinkles and bulges, averaging 4.6–8.3 × 3.2–4.6 µm (Fig 3H). Hymenophore tubes in these strains were loosely arranged, shorter (0.8–2.5 mm), and varied in diameter (0.4–1.2 mm), with regions of both broad and narrow tubes (Fig 3I-3J).For quantitative morphological measurements (cap thickness, stipe length, spore size, and hymenophore tube dimensions), 30 mature basidiocarps were randomly selected (5 biological replicates per strain, 6 strains per species) to obtain the reported size ranges. The comparison between the two morphological forms of Ganoderma is summarized in Table 3.

**Table 3:** Morphological characteristics of *Ganoderma sichuanense* (strain 41GL-5) and *Ganoderma sessile* (strain HZ-TS).

Species	Macromorphology	Microscopy	Reference
***G. sichuanense***	**Cap thickness:** 2.5–3 cm **Stipe length:** 7–11 cm (long, slender stipe)	**Spore shape:** Oval, with smooth surfaces **Spore size:** 5.6–9.2 × 3.7–5.3 µm **Hymenophore tubes**: Densely packed, slender and tubular; length 1–3 mm, diameter 0.5–1 mm (uniform), with smooth inner walls	This study
***G. sessile***	**Cap thickness:** 1.7–2.6 cm **Stipe length:** 2–4 cm (short, stout stipe)	**Spore shape**: More rounded, with noticeable surface wrinkles and bulges **Spore size:** 4.6–8.3 × 3.2–4.6 µm **Hymenophore tubes**: Loosely arranged, shorter; length 0.8–2.5 mm, diameter 0.4–1.2 mm (variable), with regions of both broad and narrow tubes	This study

### 3.3 Growth rate and cultivation performance

#### 3.3.1 Mycelial growth on PDA.

The mycelial growth rate on PDA medium is a core indicator for evaluating the vitality and adaptability of *Ganoderma* strains, which directly reflects their colonization ability under artificial culture conditions. Mycelial growth on PDA at 25 °C varied significantly among strains (Fig 4). Strains 22GL-13 (*G. sichuanense*), 41GL-2 (*G. sichuanense*), HZ-MD (*G. sessile*), and CLZ-1 (*G. sessile*) exhibited slower growth, each with an average rate below 0.5 cm/day. In contrast, strains HZ-TS (*G. sessile*), 41GL-3 (*G. sessile*), 41GL-5 (*G. sichuanense*), 22GL-12 (*G. sichuanense*), and strain LJ-1 (*G. sessile*) demonstrated faster growth, exceeding 1 cm/day. The remaining eight strains grew at intermediate rates ranging from 0.5 to 1 cm/day (excluding the two boundary values).

**Fig 4:**
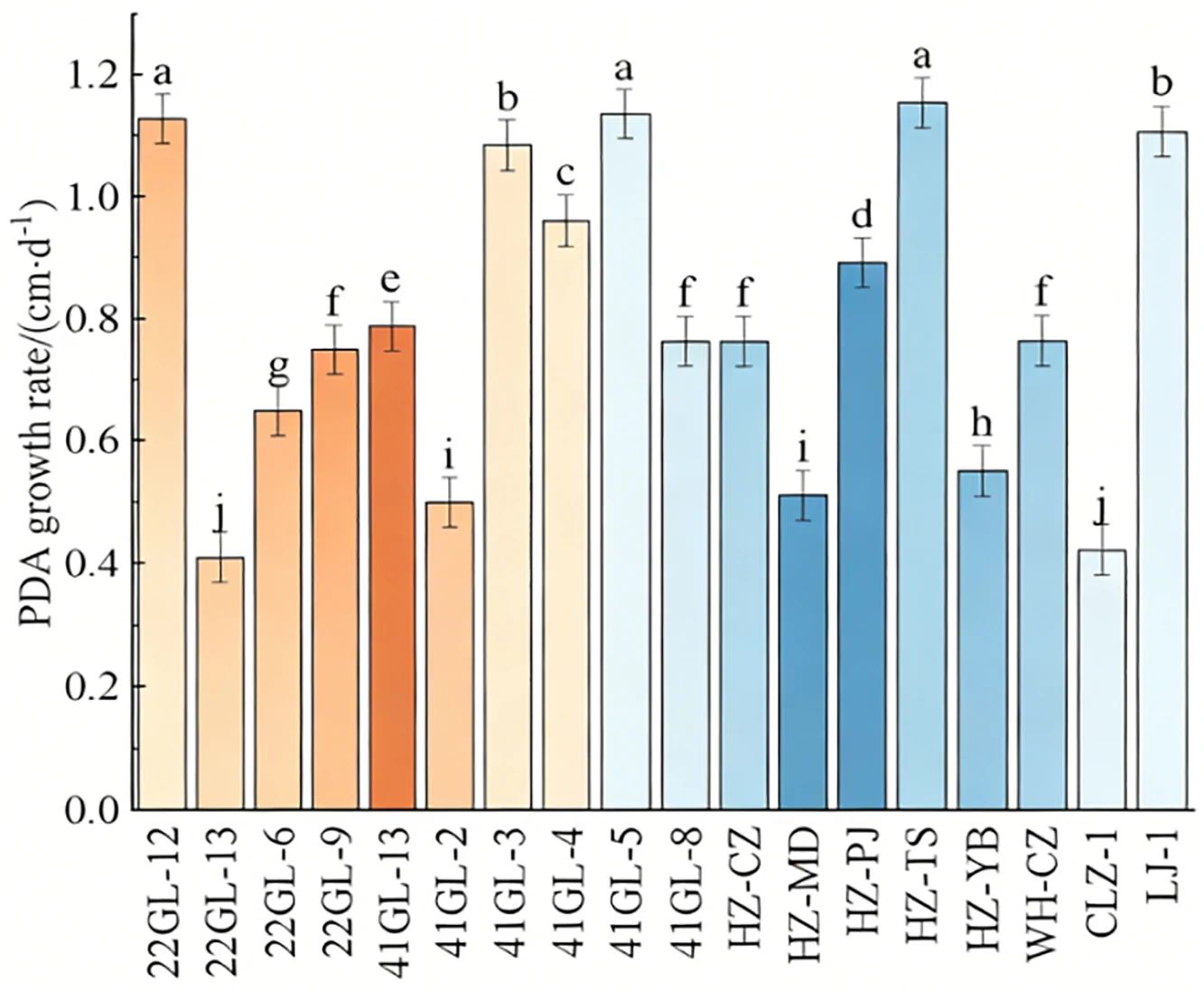
Growth performance of tested *Ganoderma* strains on PDA medium.

#### 3.3.2 Mycelial growth in cultivation substrate.

Compared with PDA-based evaluation, which primarily reflects the intrinsic growth potential of strains under controlled conditions, growth in cultivation substrate provides a more realistic assessment of their adaptability to complex nutritional environments and their practical cultivation performance. Similar patterns were observed in cultivation substrates (Fig 5). Strains HZ-TS (*G. sessile*), HZ-CZ (*G. sessile*), LJ-1 (*G. sessile*), and 22GL-6 (*G. sichuanense)* had the highest growth rates, exceeding 0.6 cm/day, with HZ-TS (*G. sessile*) showing the fastest growth overall. Other strains had lower substrate colonization rates, remaining below 0.6 cm/day.

**Fig 5:**
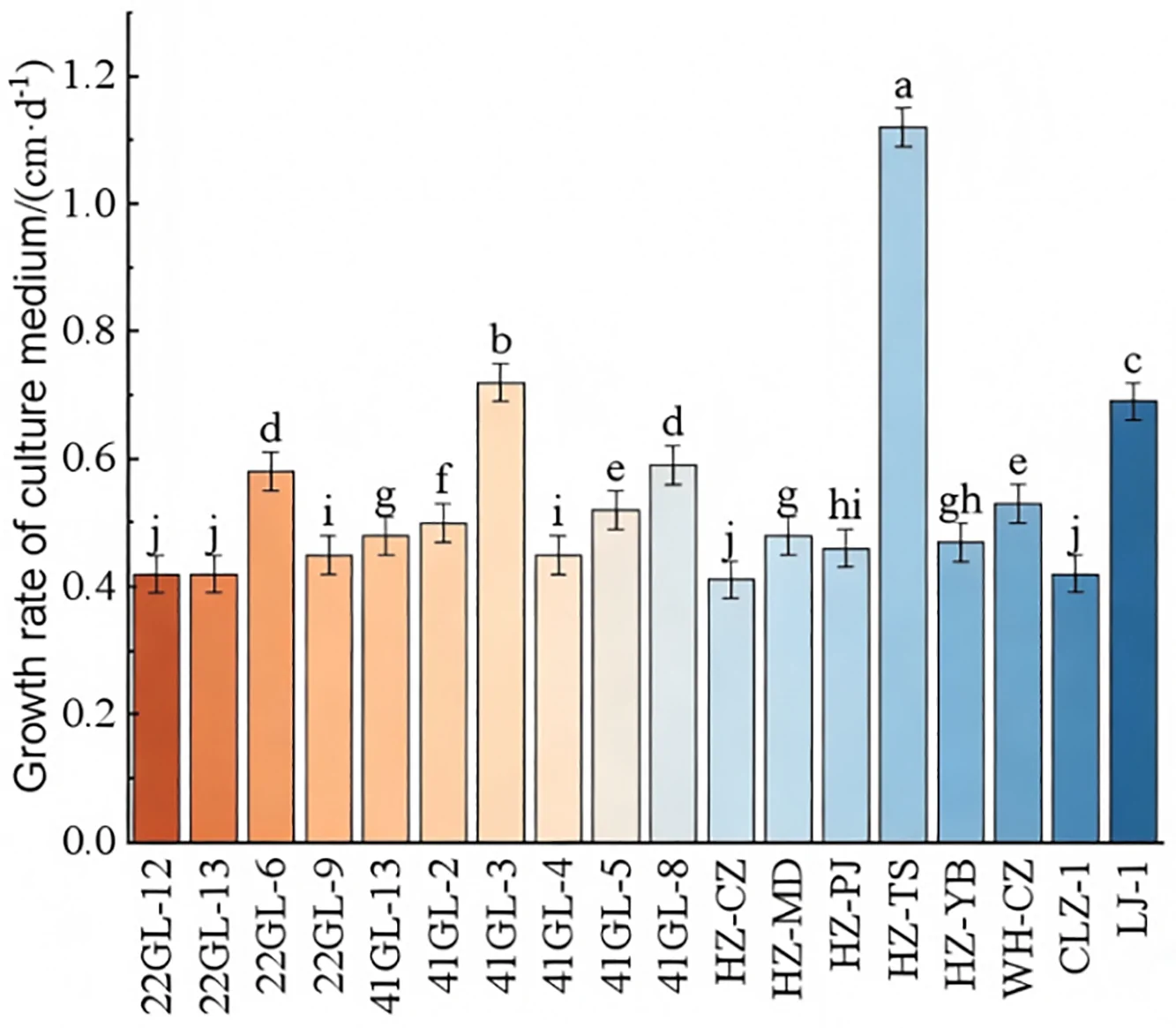
Growth performance of tested *Ganoderma* strains on substrate-based cultivation medium.

#### 3.3.3 Fruiting body production.

Fruiting body production traits, including growth cycle, morphology, and yield, are the core commercial evaluation indicators for *Ganoderma* strains, directly determining their economic value and cultivation suitability. The fruiting characteristics of the 18 *Ganoderma* strains are summarized in Table 4 and illustrated in Fig 6. Among all strains, HZ-PJ (*G. sichuanense*) initiated fruiting earliest. Several strains, including HZ-TS (*G. sessile*), 41GL-13 (*G. sichuanense*), HZ-CZ (*G. sessile*), LJ-1 (*G. sessile*), WH-CZ (*G. sichuanense*), 22GL-9 (*G. sichuanense*), HZ-CZ (*G. sichuanense*), and 41GL-8 (*G. sichuanense*), produced fruiting bodies with cap diameters exceeding 10 cm, while the remaining strains produced smaller caps (<10 cm). In terms of cap thickness, 41GL-13 (*G. sichuanense*), LJ-1 (*G. sessile*), 22GL-6 (*G. sichuanense*), WH-CZ (*G. sichuanense*), 22GL-9 (*G. sichuanense*), 41GL-2 (*G. sichuanense*), HZ-PJ (*G. sichuanense*), 22GL-12 (*G. sichuanense*), HZ-MD (*G. sessile*), and HZ-CZ (*G. sichuanense*) exceeded 9 cm, with 22GL-9 (*G. sichuanense)*, 41GL-2 (*G. sichuanense*), and HZ-CZ (*G. sichuanense*) surpassing 11 cm. High fruiting body yield was observed in strains 41GL-13 (*G. sichuanense*), HZ-CZ (*G. sessile*), 22GL-6 (*G. sichuanense*),WH-CZ (*G. sichuanense*), HZ-PJ (*G. sichuanense*), 22GL-12 (*G. sichuanense*), and HZ-CZ (*G. sichuanense*), each producing more than 30 g/bag of dry weight. Notably, 22GL-12 (*G. sichuanense*) had the highest yield at 52 g/bag, followed by HZ-PJ (*G. sichuanense*) (39 g/bag) and 22GL-6 (34 g/bag.

**Table 4:** Morphological characteristics of the 18 tested *Ganoderma* strains.

Strain	Primordium formation time(d)	Pileus			Stem			Dry weight (g)
		L (cm)	Wide (cm)	Thick (cm)	L (cm)	Dia (cm)	Branch	
HZ-TS (*G. sessile*)	22.0 ± 0.8	12.5 ± 0.6	7.0 ± 0.4	10.9 ± 0.5	6.5 ± 0.3	1.2 ± 0.1	2.0 ± 0.2	18.2 ± 1.3
41GL-13 (*G. sichuanense)*	24.0 ± 0.9	14.0 ± 0.7	7.5 ± 0.4	10.3 ± 0.5	3.0 ± 0.2	0.7 ± 0.1	2.0 ± 0.2	32.2 ± 2.1
41GL-3 (*G. sessile*)	19.0 ± 0.7	11.5 ± 0.6	6.5 ± 0.3	7.8 ± 0.4	13.0 ± 0.6	0.6 ± 0.1	2.0 ± 0.2	31.2 ± 2.0
LJ-1 (*G. sessile*)	18.0 ± 0.6	12.5 ± 0.6	9.0 ± 0.5	7.3 ± 0.4	4.0 ± 0.2	0.6 ± 0.1	2.0 ± 0.2	23.6 ± 1.6
22GL-6 (*G. sichuanense*)	18.0 ± 0.6	6.5 ± 0.3	6.0 ± 0.3	9.4 ± 0.5	6.5 ± 0.3	1.6 ± 0.1	3.0 ± 0.2	34.0 ± 2.3
WH-CZ (*G. sichuanense*)	19.0 ± 0.7	11.5 ± 0.6	7.0 ± 0.4	8.2 ± 0.4	9.5 ± 0.5	1.4 ± 0.1	1.0 ± 0.1	31.0 ± 2.0
41GL-5 (*G. sichuanense*)	18.0 ± 0.6	9.7 ± 0.5	5.7 ± 0.3	5.8 ± 0.3	12.0 ± 0.6	1.1 ± 0.1	1.0 ± 0.1	27.0 ± 1.8
22GL-9 (*G. sichuanense*)	22.0 ± 0.8	13 ± 0.6	8.0 ± 0.4	10.4 ± 0.5	7.0 ± 0.3	1.6 ± 0.1	1.0 ± 0.1	22.0 ± 1.5
41GL-2 (*G. sichuanense*)	37.0 ± 1.2	11 ± 0.5	8.0 ± 0.4	9.9 ± 0.5	7.0 ± 0.3	1.1 ± 0.1	1.0 ± 0.1	28.0 ± 1.9
HZ-PJ (*G. sichuanense*)	15.0 ± 0.5	9.5 ± 0.5	6.5 ± 0.3	7.3 ± 0.4	17.0 ± 0.8	1.1 ± 0.1	2.0 ± 0.2	39.0 ± 2.6
22GL-12 (*G. sichuanense*)	25.0 ± 0.9	9.0 ± 0.4	5.0 ± 0.3	9.8 ± 0.5	12.0 ± 0.6	2.0 ± 0.1	2.0 ± 0.2	52.0 ± 3.4
HZ-MD (*G. sessile*)	23.0 ± 0.8	5.5 ± 0.3	4.5 ± 0.2	7.5 ± 0.4	9.0 ± 0.4	1.1 ± 0.1	3.0 ± 0.2	27.0 ± 1.8
HZ-CZ (*G. sichuanense*)	25.0 ± 0.9	11.0 ± 0.5	7.5 ± 0.4	9.5 ± 0.5	10.0 ± 0.5	1.7 ± 0.1	1.0 ± 0.1	33.0 ± 2.2
41GL-8 (*G. sichuanense*)	18.0 ± 0.6	10.0 ± 0.5	6.0 ± 0.3	7.0 ± 0.4	11.5 ± 0.6	1.2 ± 0.1	2.0 ± 0.2	29.0 ± 1.9
CLZ-1 (*G. sessile*)	36.0 ± 1.2	8.0 ± 0.4	4.5 ± 0.2	6.0 ± 0.3	6.5 ± 0.3	1.0 ± 0.1	3.0 ± 0.2	26.0 ± 1.7
41GL-4 (*G. sessile*)	24.0 ± 0.8	7.0 ± 0.3	5.0 ± 0.3	7.6 ± 0.4	19 ± 0.8	1.4 ± 0.1	2.0 ± 0.2	25.0 ± 1.7
HZ-YB (*G. sessile*)	37.0 ± 1.3	11.5 ± 0.6	8.5 ± 0.4	8.2 ± 0.4	5.5 ± 0.3	1.3 ± 0.1	2.0 ± 0.2	24.5 ± 1.6
22GL-13 (*G. sessile*)	26.0 ± 1.0	11.7 ± 0.6	6.9 ± 0.4	8.8 ± 0.4	11.3 ± 0.5	1.5 ± 0.1	1.0 ± 0.1	31.5 ± 2.0

**Note:** Primor: Primary morphological characters; L: Length; W: Width; T: Thickness; Dia: Diameter; Branch: Branching number; Dry wt (g): Dry weight (grams). Values are mean ± standard deviation (SD) of 15 replicate bags per strain.

**Fig 6:**
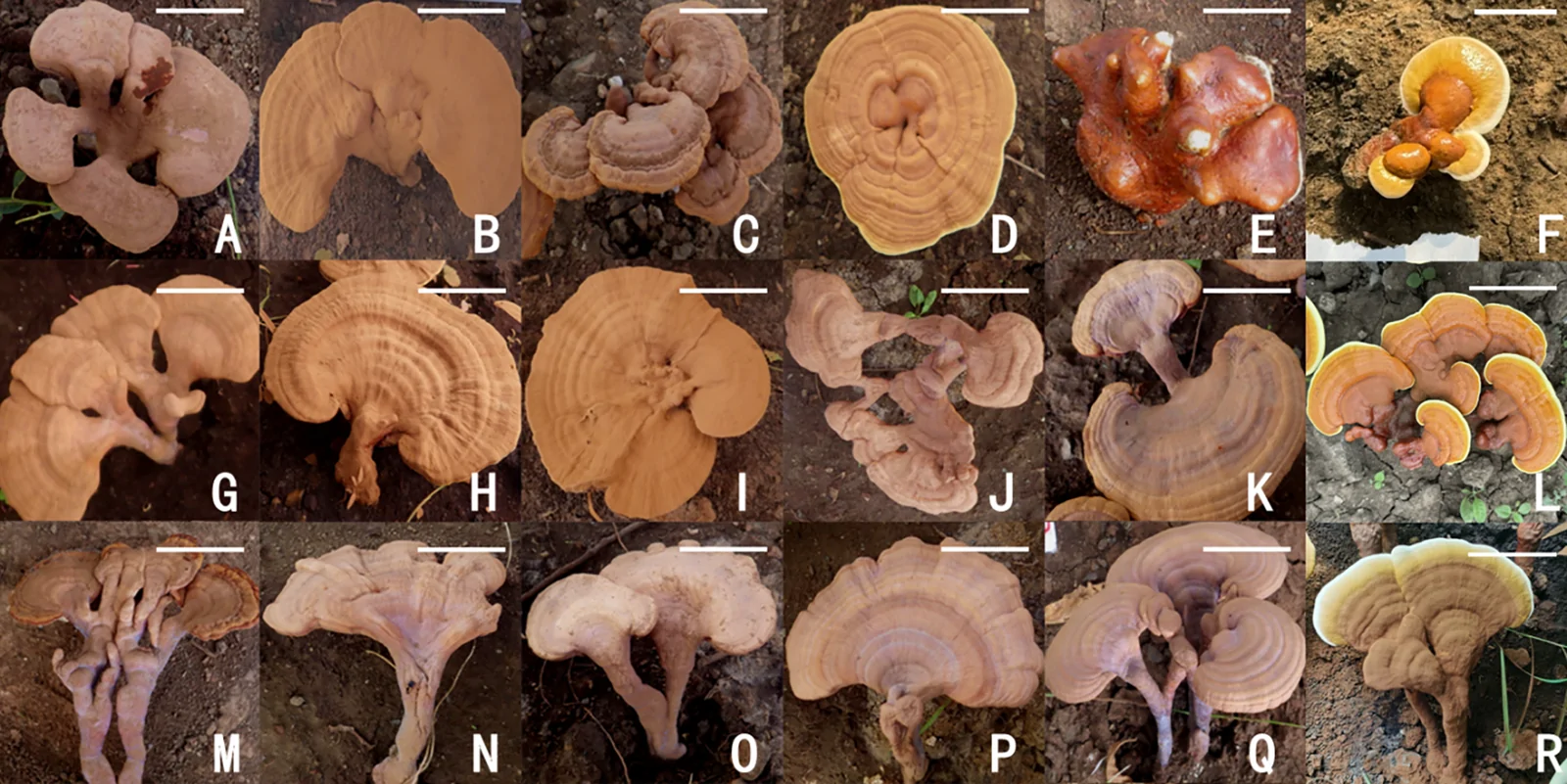
Fruiting body morphology of selected strains. A: 22GL-6 (*G. sichuanense*); B:41GL-3 (*G. sessile*); C: 41GL-4 (*G. sessile*); D:41GL-13 (*G. sichuanense*); E:HZ-YB (*G. sessile*); F:HZ-MD (*G. sessile*) G: HZ-CZ (*G. sichuanense*); H:HZ-TS (*G. sessile*); I:LJ-1 (*G. sessile*); J:22GL-12 (*G. sichuanense*); K: 41GL-8 (*G. sichuanense*); L:22GL-13 (*G. sichuanense*); M: HZ-PJ (*G. sichuanense*); N: WH-CZ (*G. sichuanense*); O: 41GL-5 (*G. sichuanense*); P: 22GL-9 (*G. sichuanense*); Q: CLZ-1 (*G. sichuanense*); R: 41GL-2 (*G. sichuanense*). Bars = 4 cm.

### 3.4 Bioactive compound analysis of fruiting bodies

#### 3.4.1 Polysaccharide content.

Polysaccharides are among the most studied bioactive components in *Ganoderma*, with well-documented immunomodulatory and anti-tumor activities. Their content is therefore a key marker for evaluating the functional quality of different strains. Polysaccharide content varied significantly among the 18 strains, as shown in Fig 7, all experiments were performed with three independent biological replicates. The highest concentration was found in HZ-PJ (*G. sichuanense*) at 12.72 mg/g, followed by 41GL-2 (*G. sichuanense*) and 41GL-4 (*G. sessile*). Intermediate levels (approximately 8.10 mg/g) were observed in strains such as WH-CZ (*G. sichuanense*), 41GL-5 (*G. sichuanense*), 41GL-8 (*G. sichuanense*), 22GL-6 (*G. sichuanense*), HZ-CZ (*G. sichuanense*), HZ-TS (*G. sessile*), and 41GL-13 (*G. sichuanense*). In contrast, strains HZ-YB(*G. sessile*), LJ-1 (*G. sessile*), 41GL-3 (*G. sessile*), 22GL-13 (*G. sessile*), 22GL-9 (*G. sichuanense*), 22GL-12 (*G. sichuanense*), HZ-MD (*G. sessile*), and CLZ-1 (*G. sessile*) showed relatively low polysaccharide content, ranging from 3.99 to 5.12 mg/g.

**Fig 7:**
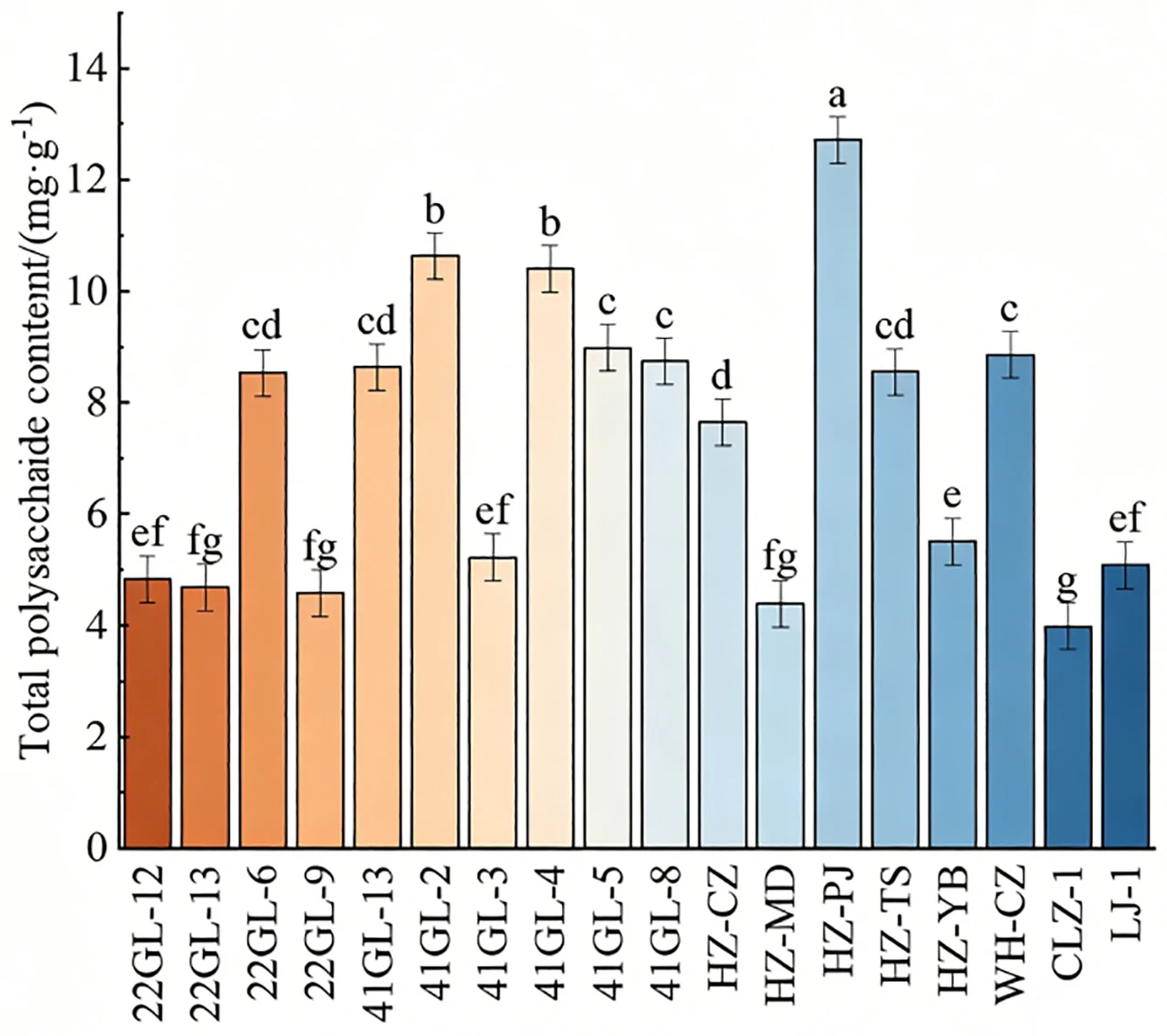
Comparison of polysaccharide content of tested *Ganoderma* strains.

#### 3.4.2 Antioxidant capacity.

Antioxidant activity is a key functional trait of *Ganoderma* relevant to its nutraceutical value, and multiple assays enable comprehensive evaluation of this property. Antioxidant activities of the fruiting bodies were assessed using both ABTS and DPPH assays (Fig 8), all experiments were performed with three independent biological replicates. Several strains, including WH-CZ (*G. sichuanense*), HZ-YB (*G. sessile*), 41GL-2 (*G. sichuanense*), 22GL-13 (*G. sessile*), 41GL-5 (*G. sichuanense*), 41GL-3 (*G. sessile*), 22GL-9 (*G. sichuanense*), HZ-MD (*G. sessile*), 41GL-4 (*G. sessile*), HZ-CZ (*G. sichuanense*), and LJ-1 (*G. sessile*), demonstrated strong antioxidant activity in the ABTS assay.

**Fig 8:**
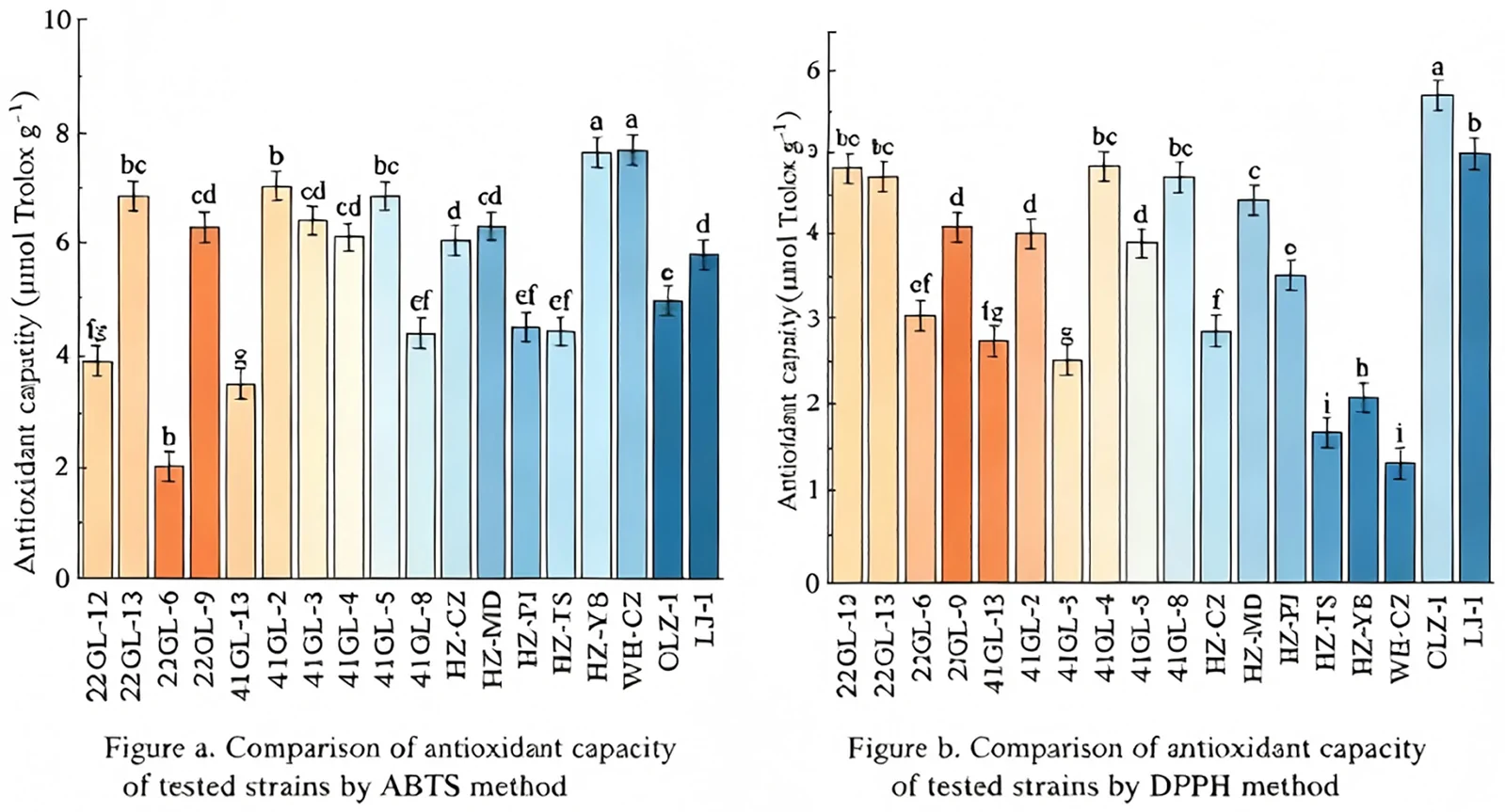
Comparison of antioxidant capacity of tested *Ganoderma* strains.

In the DPPH assay, high antioxidant capacity was detected in CLZ-1 (*G. sessile*), LJ-1 (*G. sessile*), 22GL-12 (*G. sichuanense*), 41GL-4 (*G. sessile*), 22GL-13 (*G. sessile*), 41GL-8 (*G. sichuanense*), HZ-MD (*G. sessile*), 22GL-*9* (*G. sichuanense*), 41GL-2 (*G. sichuanense*), 41GL-5 (*G. sichuanense*), and HZ-PJ (*G. sichuanense*). Notably, strains 22GL-13 (*G. sessile*), 41GL-2 (G*. sichuanense*), 41GL-5 (*G. sichuanense*), 22GL-9 (*G. sichuanense*)*,* LJ-1 (*G. sessile*), and HZ-MD (*G. sessile*) exhibited strong antioxidant activity in both assays, highlighting the value of multi-method approaches to accurately evaluate antioxidant potential.

#### 3.4.3 Flavonoids content.

Flavonoids are polyphenols with antioxidant and anti‑inflammatory bioactivities, and their contents in *Ganoderma* are vital for functional food exploitation. Flavonoid content across the 18 strains is presented in Fig 9, all experiments were performed with three independent biological replicates. Under uniform cultivation conditions, strains LJ-1 (*G. sessile*), CLZ-1(*G. sessile*), HZ-TS (*G. sessile*), 22GL-12 (*G. sichuanense*), WH-CZ (*G. sichuanense*), 41GL-3 (*G. sessile*), and (*G. sichuanense*) exhibited notably high levels of flavonoids. Given the known antioxidant and health-promoting properties of flavonoids, these strains may have particular value for use in nutraceuticals and functional foods.

**Fig 9:**
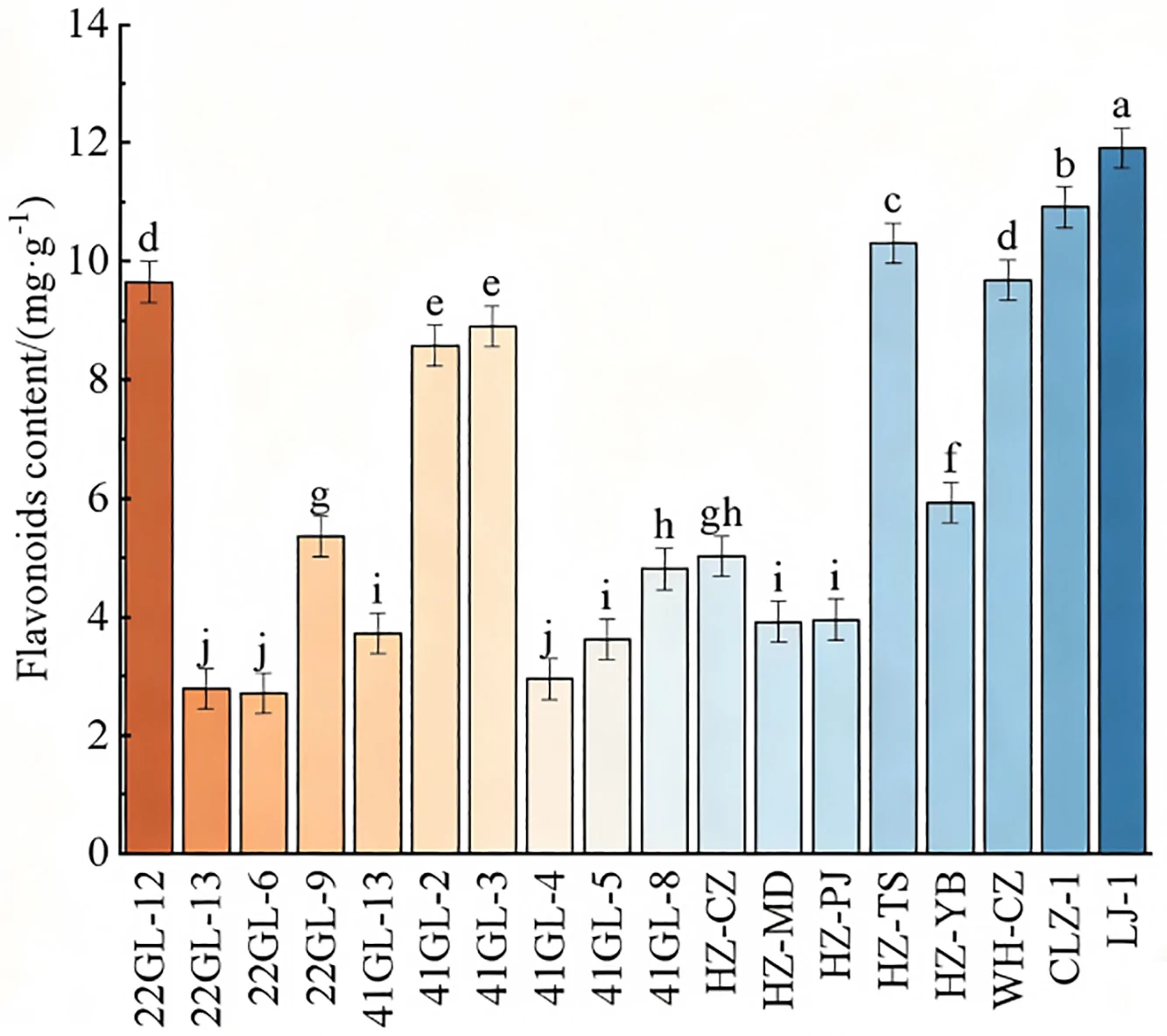
Flavonoids content of the tested *Ganoderma* strains.

#### 3.4.4 Triterpenes content.

Triterpenes are characteristic bioactive compounds of *Ganoderma*. The triterpene concentrations in the fruiting bodies of the 18 *Ganoderma* strains are shown in Fig 10, all experiments were performed with three independent biological replicates. Under standardized cultivation conditions, several strains exhibited notably high triterpene levels. Specifically, strains with notably high triterpene contents (exceeding 5 mg/g) were, in descending order: HZ-TS (*G. sessile*), LJ-1 (*G. sessile*), CLZ-1 (*G. sessile*), 41GL-2 (*G. sichuanense*), 22GL-9 (*G. sichuanense*), 22GL-12 (*G. sichuanens*e), and HZ-YB (*G. sessile*). Among them, HZ-TS (*G. sessile*) showed the highest triterpene content at 12.2 mg/g, which was significantly higher than that of the other strains.

**Fig 10:**
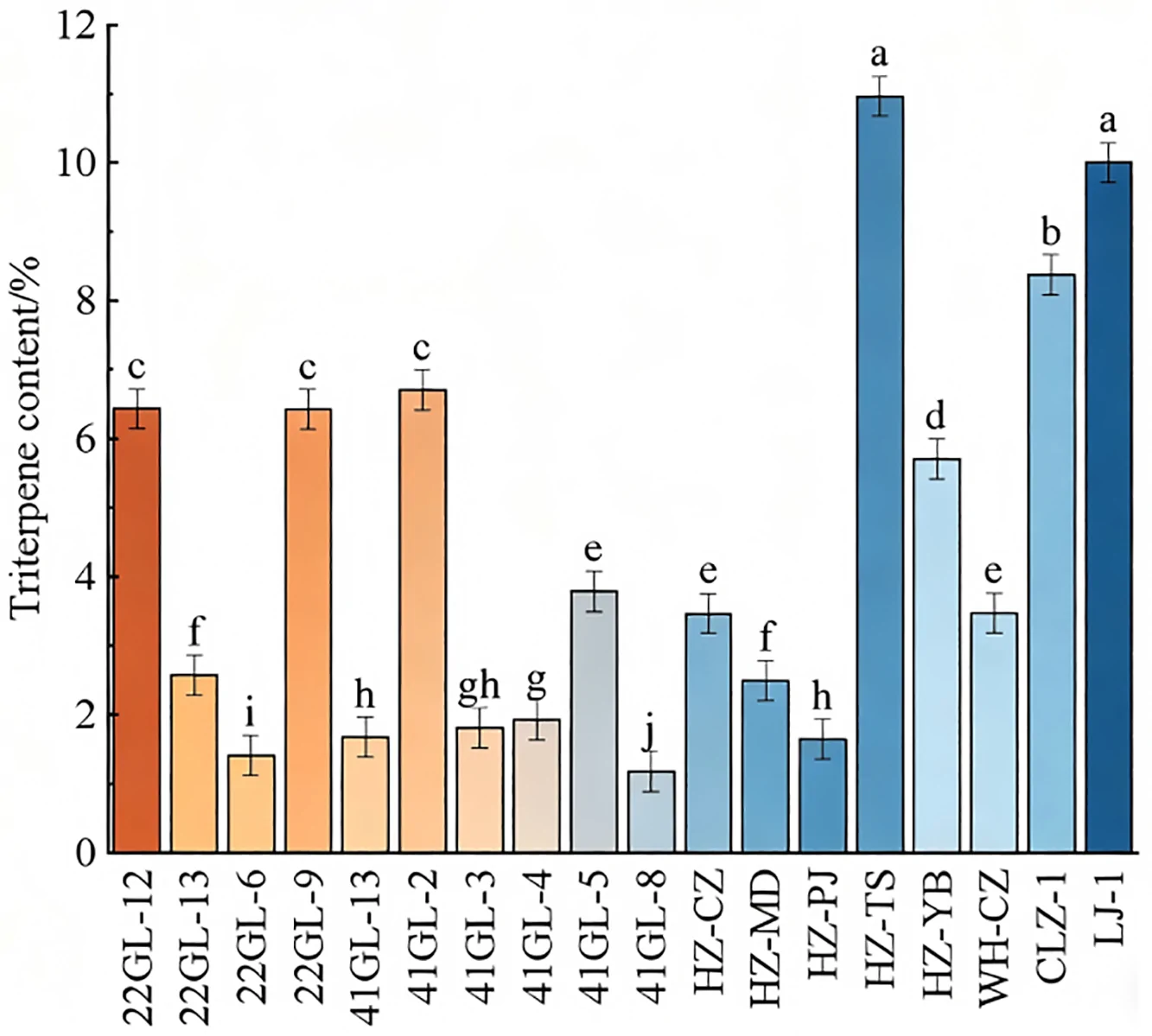
Triterpene content analysis of the tested *Ganoderma* strains.

### 3.5 Trace elements and heavy metals in fruiting bodies

Trace element and heavy metal contents determine the nutritional quality and edible safety of *Ganoderma* fruiting bodies. The contents of four metallic elements (cadmium, lead, potassium and zinc) were determined across the 18 tested *Ganoderma* strains, all experiments were performed with three independent biological replicates, and elemental uptake profiles varied notably among isolates, as summarized in Fig 11. While the overall trends in metal accumulation were relatively consistent across strains, notable quantitative differences were identified.

**Fig 11:**
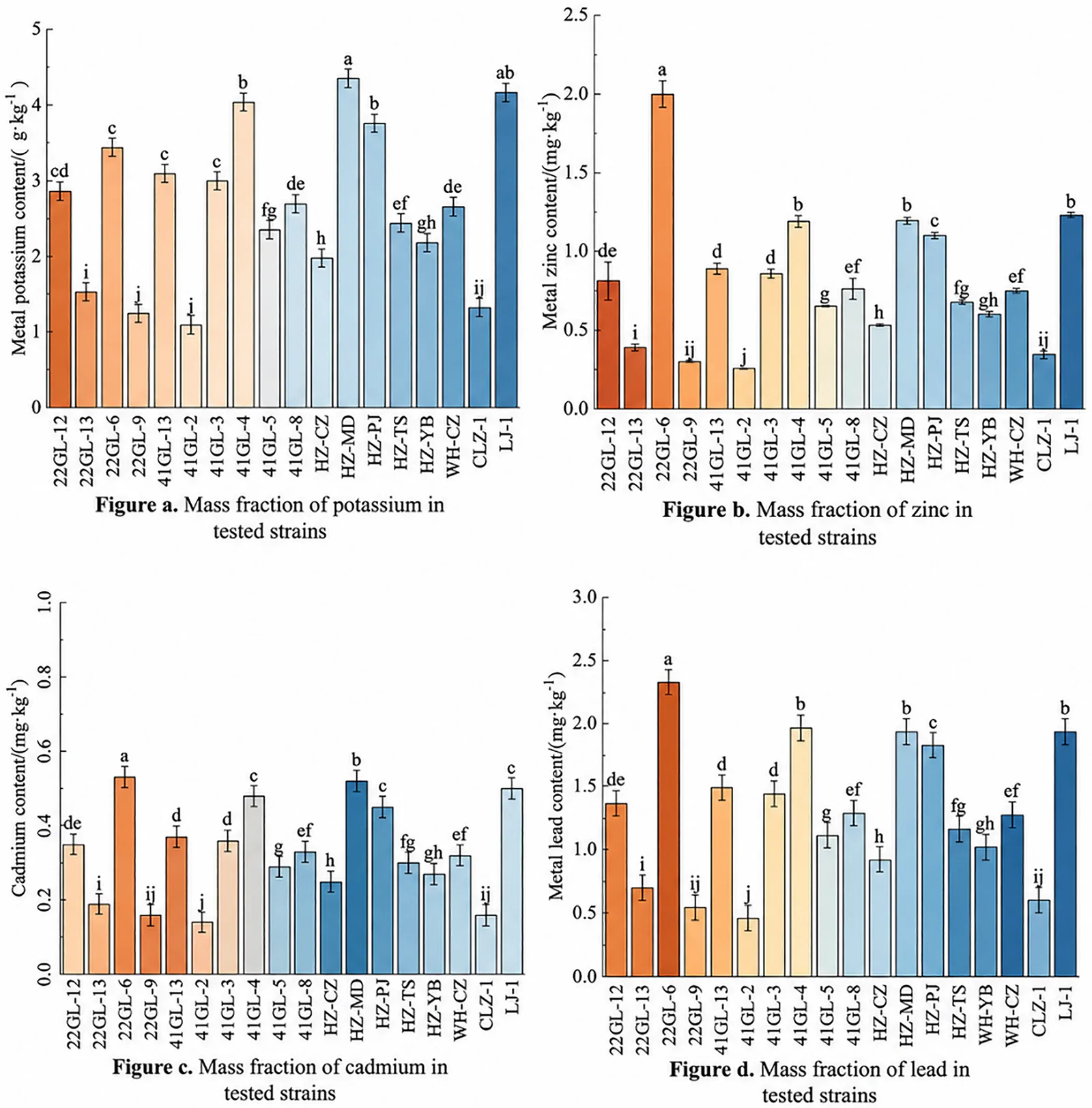
Concentrations of trace elements and heavy metals in fruiting bodies of 18 tested *Ganoderma* strains.

The accumulation of individual elements varied considerably among strains: Potassium (K) concentrations were highest in strain HZ-MD (4.4 g·kg ^-1^), followed by strains 41GL-4 (4.1 g·kg^-1^) and LJ-1 (4.2 g·kg ^-1^); Zinc (Zn) levels were most abundant in strains 22GL-12 (2.0 mg·kg^-1^) and LJ-1 (1.2 mg·kg^-1^). Cadmium (Cd) concentrations of all strains ranged from 0.13 to 0.55 mg·kg^-1^, with the highest levels observed in strains 22GL-6 (0.55 mg·kg^-1^) and HZ-PJ (0.52 mg·kg^-1^); Lead (Pb) levels ranged from 0.4 to 2.2 mg·kg^-1^, peaking at 2.2 mg·kg^-1^ in strain 22GL-6.

To assess food safety, these heavy metal concentrations were compared with the maximum permissible limits specified in the Chinese national food safety standard GB 2762−2022 for edible fungi (Cd ≤ 0.5 mg·kg^-1^, Pb ≤ 1.0 mg·kg^-1^). Most strains met the safety requirements for both Cd and Pb. However, a few strains, including 22GL-6, exhibited slightly elevated Pb levels that exceeded the standard limit, while Cd concentrations remained within the acceptable range for all strains. These results highlight the variability in elemental uptake among different *Ganoderma* strains and underscore the importance of careful strain selection for food and medicinal applications to ensure both safety and nutritional quality.

## 4. Discussion

In this study, 18 widely cultivated *Ganoderma* strains from different provinces across China were subjected to comprehensive morphological, phylogenetic, and biochemical analyses. By linking species identity with functional traits, our results move beyond descriptive analysis and directly inform strain selection and production management.

Phylogenetic analysis based on combined ITS, RPB2, and TEF1-α sequences revealed that among the 18 tested strains, 11 belonged to *G. sichuanense* and 7 belonged to *G. sessile*. This confirms the coexistence of these two species in commercial *Ganoderma* cultivation systems in China, which is consistent with previous reports indicating cryptic diversity within the *G. lucidum* species complex [14,28]. Morphological observations further supported the phylogenetic classification: *G. sichuanense* exhibited thicker pilei (2.5–3 cm), longer stipes (7–11 cm), oval and smooth basidiospores, and densely arranged hymenophore tubes; in contrast, *G. sessile* had thinner pilei (1.7–2.6 cm), shorter stipes (2–4 cm), rounded spores with surface wrinkles, and loosely arranged hymenophore tubes. These morphological distinctions are consistent with the original descriptions of these two species and provide practical tools for preliminary identification in the absence of molecular resources [14].

From a cultivation perspective, this distinction is critical because species-level differences translated into clear phenotypic divergence in our study. *G. sessile* strains showed shorter growth cycles, making them suitable for fast-cropping production and regions with limited growing seasons. In contrast, *G. sichuanense* strains displayed superior yield-related traits, demonstrating greater potential for high-yield cultivation under stable environmental conditions. These results highlight that accurate species identification is not only taxonomically important but also essential for matching strains with appropriate cultivation strategies [32].

Growth performance analysis revealed that strain adaptability is strongly genotype-dependent. A key outcome of this study is the discrepancy observed between PDA-based growth rates and substrate colonization ability. While PDA assays are widely used for rapid evaluation, our results demonstrate that they do not always predict performance under cultivation conditions. For example, strains such as HZ-TS maintained high growth rates in both PDA and substrate systems, indicating stable adaptability and making them strong candidates for commercial cultivation. In contrast, other strains showed inconsistent performance, emphasizing that substrate-based evaluation is essential before large-scale application. This finding aligns with previous studies showing that substrate complexity and environmental interactions significantly influence fungal growth [45]. Therefore, cultivation strategies should prioritize strains that exhibit stable performance across both laboratory and substrate conditions.

Yield-related traits further demonstrated that high mycelial growth does not necessarily translate into high productivity. In our results, high-yielding strains (e.g., 22GL-12 and HZ-PJ) did not always correspond to the fastest-growing strains, indicating that yield is regulated by multiple physiological processes, including nutrient allocation and reproductive efficiency. This is consistent with general principles in mushroom cultivation, where yield depends on both genetic potential and environmental conditions [46].

Biochemical analysis revealed clear functional differentiation between strains and species. Notably, the polysaccharide content of the elite *G. sichuanense* strain HZ-PJ reached 12.72 mg/g under standardized artificial cultivation. According to published data for conventionally cultivated *Ganoderma* fruiting bodies, total polysaccharide contents generally range from 6.0 to 13.0 mg/g under comparable substrate and climatic conditions [47]. Therefore, the 12.72 mg/g polysaccharide level detected in HZ-PJ represents a relatively high, typically superior level for commercially cultivated *Ganoderma*, exceeding typical values and indicating strong genetic potential for polysaccharide biosynthesis, and confirming that HZ-PJ is a superior germplasm for polysaccharide-targeted functional products. In contrast, *G. sessile* strain HZ-TS accumulated higher triterpene and flavonoid contents, which is consistent with the typical metabolic characteristics of *G. sessile* reported in previous studies. Such genotype-driven metabolic divergence enables precise industrial positioning: high-polysaccharide *G. sichuanense* strains are more suitable for immunomodulatory health-care products, while triterpene-enriched *G. sessile* strains have greater commercial value for antioxidant and pharmaceutical applications [48,49].

The analysis of trace elements and heavy metals adds an important dimension to cultivation strategy by addressing safety considerations. Despite identical cultivation conditions, significant differences in element accumulation were observed among strains, indicating that metal uptake is largely genotype-dependent. This is consistent with previous reports on edible fungi [50]. From a practical perspective, selecting strains with low heavy metal accumulation is essential for ensuring compliance with food and medicinal safety standards. Incorporating such screening into strain evaluation protocols can reduce risks associated with contamination and improve product quality.

Despite the multi-dimensional evaluation conducted in this study, several limitations remain. First, all experiments were performed under relatively controlled conditions; thus, strain performance under diverse field environments and different substrate formulas requires further verification [51,52]. Second, although phenotypic differences in growth, yield, and metabolite accumulation were clearly characterized, the underlying genetic and molecular regulatory mechanisms remain unclear. Future multi-omics studies combining genomics, transcriptomics, and metabolomics will help elucidate key metabolic pathways and regulatory genes responsible for elite agronomic and safe traits.

For industrial application, future *Ganoderma* cultivation should adopt precision and goal-oriented management. Fast-cycle strains can be applied for rapid production, while high-polysaccharide or high-triterpene strains can be selectively cultivated for specific functional and medicinal markets. Integrating phenotypic superiority, active ingredient accumulation, and heavy metal safety into systematic strain screening will benefit standardized and sustainable *Ganoderma* production [53]. Overall, further exploration of germplasm resources, mechanistic dissection of core agronomic traits, and optimization of targeted cultivation strategies will continuously promote the industrial upgrading of *Ganoderma* cultivation [54].

## 5. Conclusion

This study provided a systematic evaluation of 18 *Ganoderma* strains cultivated across diverse regions in China. Both *G. sichuanense* and *G. sessile* were found to coexist in cultivation environments, though further phylogenetic refinement is needed to validate the classification of several *G. sessile* strains.

Among the tested strains, HZ-PJ (*G. sichuanense*) exhibited favorable comprehensive traits, combining early fruiting, high yield, and superior polysaccharide and antioxidant levels. Similarly, strain HZ-TS (*G. sessile*) showed promise due to its high yield, elevated levels of triterpenes and flavonoids, and stable growth performance across both laboratory and substrate conditions. These two strains represent strong candidates for commercial cultivation, especially in functional food and medicinal mushroom markets, as their traits align clearly with specific product development goals.

Critically, our results also revealed significant genotypic variation in heavy metal accumulation among strains grown under identical conditions. This finding highlights that safety risks are not only environmental but also strain-dependent, making genotype selection a key tool to meet food and medicinal safety standards. Incorporating heavy metal screening into routine strain evaluation protocols can directly reduce contamination risks and improve the reliability of commercial products.

In summary, this study demonstrates that accurate species identification, combined with multi-trait evaluation of growth, yield, bioactive compound profiles, and heavy metal accumulation, provides a robust framework for evidence-based strain selection. These findings are directly applicable to real-world production systems, enabling producers to match strains with local climate conditions, cultivation cycles, and product objectives while ensuring compliance with safety regulations. Further research should focus on optimizing cultivation protocols for these promising strains, elucidating the genetic mechanisms underlying their advantageous traits, and developing simple, scalable screening tools to support widespread adoption in commercial settings [53,54].
